# Political turnover and corporate financialization: Evidence from China

**DOI:** 10.3389/fpsyg.2022.1039560

**Published:** 2022-10-26

**Authors:** Simeng Lyu, Yong Qi, Shuo Yang, Shaoyu Dong

**Affiliations:** School of Business Administration, Northeastern University, Shenyang, China

**Keywords:** D72 D78 G32 G38, political turnover, corporate financialization, fixed asset investment, cash holdings, from virtual to real

## Abstract

In the context of the slowing growth of the real economy and the rapid development of the financial industry, more and more non-financial companies are participating in the financial industry for the purpose of development and profit expansion. China has gradually appeared the phenomenon of corporate financialization. This paper uses the panel fixed effect model empirically examines the effect of political turnover on corporate financialization by using data of listed companies and top prefecture level officials in China between 2007 and 2020. We find that the turnover of mayors significantly decreases corporate financialization, while the turnover of party secretaries has no impact on corporate financialization. Moreover, these results are moderated by the characteristics of government officials and firm’s characteristics. Our results further show that changes by mayors increase fixed asset investment and decrease cash holdings, and, thus, reduce corporate financialization. These findings could assist in solving the “from real to virtual” problems, strengthening financial services, and realizing high-quality economic development.

## Introduction

Since the 1980s, the return rate of Chinese traditional industry companies has significantly reduced because of the shrinking market demand, and overcapacity in the real economy. Further, the virtual economy has accelerated expansion and many industrial capitals have been poured into financial and real estate industries with higher rates of return. This typically leads to corporate financialization, which existing literature suggests being closely related to economic globalization and economic crises (Tori and Onaran, 2018; Wang, 2019).

**Figure 1:**
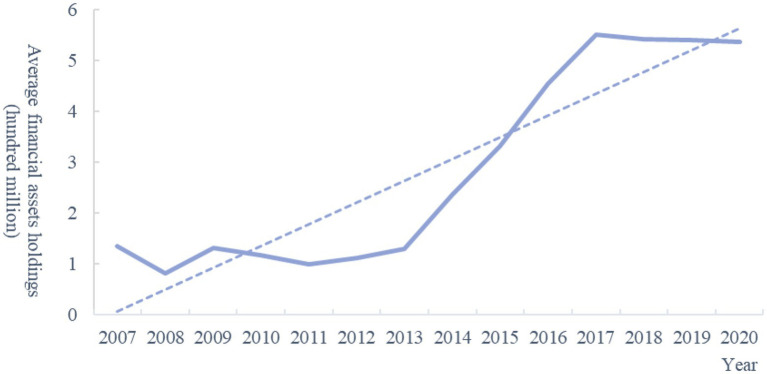
Average financial assets holdings of non-financial companies in China. Data collected from CSMAR (China Stock Market and Accounting Research Database) database and calculated by authors. The specific types of financial assets are detailed in the manuscript.

Recently, the phenomenon of corporate financialization in China has attracted extensive attention as many non-financial companies devote themselves to the financial industry to improve profitability, and deviate from main business development. According to Figure 1, the average financial assets holdings of China’s non-financial companies averaged 136 million in 2007, followed by a wave of growth, reaching 536 million by 2020. It can also be seen from the trend line that, on average, the scale of financial assets holdings by listed companies shows an obvious upward trend. The 19th National Congress of the Communist Party of China emphasized that the focus of economic development should be on the real economy.

Political turnover, which refers to the resignation of senior leadership positions, is generally considered to be the major decisive factor of economic growth (Jones and Olken, 2005). Previous research shows that frequent officials turnover disrupts the allocation of resources and induces uncertainties, which is damaging to economic development (Earle and Gehlbach, 2015). Zhou et al. (2015) explain from the perspective of political promotion tournaments why the jurisdiction officials are so eager to construct infrastructure and helping local enterprises under the risk of overuse of resources. Political turnover is a critical and dynamic aspect of acknowledging local government behavior, as well as a new frontier for investigating the features of local government in stimulating local economic growth. However, the impact of political factors on corporate financialization have not been found.

China provides an ideal laboratory environment to analyze the impact of political turnover on corporate financialization for the following aspects. Firstly, political promotion is the single way for outstanding politicians to promote to a higher office under China’s political system. In addition, the central government of China has attached great importance to solving the “from real to virtual” problem to ensure that the financial market better serves the real economy. This is because over-financialization may generate or aggravate financial risk (Barradas and Lagoa, 2017) and trigger a financial crisis (Palley, 2013).

In this study, we accumulate the information of 995 mayors and 912 party secretaries from 2007 to 2020 period and combine it with firm data to examine how NFCs financialization is affected by political turnover. Moreover, we also make a distinction between different types of officials. First, since the promotion of outsiders will lead to greater changes in economic policy, we classify political turnover as either external promotions or local appointments. We assume that corporate financialization will decline more significantly with external appointments. Furthermore, since the age of appointed officials is an important consideration in the evaluation of political promotion under the compulsory retirement system—there are relatively higher promotion possibilities for younger politicians and appointments of young officials arouse to more uncertainty in local firms—we suppose that younger officials lead to lower corporate financialization. In additional tests, we examine whether firms that are non-state-owned and small-sized exhibit lower corporate financialization during political turnover and whether fixed asset investment and internal cash holdings play a mediating role between political turnover and corporate financialization.

This article contributes in three aspects. First, we advance the literature on the economic consequences of political turnover at firm level by focusing on the effects of corporate financialization. Moreover, we conduct more detailed research on the heterogeneous of the new officials and corporate features, making the research more reliable. Finally, we establish a “political turnover—fixed asset investment and cash holdings—corporate financialization” theoretical analysis framework, which empirically tests the influence mechanism of political turnover on corporate financialization. Previous literature demonstrates that political turnover influence corporate innovation, investment and risk-taking (An et al., 2016; Bhattacharya et al., 2017; Luo et al., 2017). However, how political turnover influences corporate financialization has remained unclear.

The remainder of this paper is organized as follows. “Literature review and research hypothesis” briefly describes the literature review and research hypothesis. “Materials and methods” presents the methods. “Results” provides empirical evidence of the relationship between political turnover and corporate financialization. “Heterogeneity of official and companies” presents the heterogeneity analysis. “Mediating effect” presents the results of the mediating effect. “Conclusion and implications” presents the conclusions and policy implications.

## Literature review and research hypothesis

### Literature review

Corporate financialization is increasingly considered a crucial business strategy by researchers and investors (Orhangazi, 2008), and the existing research mainly focus on the follow three aspects. The first is the definition of corporate financialization. Krippner (2005) demonstrates that financialization refers to the profit accumulation mainly by financial investments rather than fixed asset investments. The second is the motivation for financial assets, which can be attributed to two aspects: precautionary savings and profit pursuit. Precautionary savings refers to the allocation of low-risk financial assets based on liquidity savings in response to uncertainty about future policy, and operating cash flow rupture. When there are potential investment opportunities of financial difficulties, firms can quickly release financial assets to supplement liquidity and relieve capital pressure, especially for firms with financing constraints (Denis and Sibilkov, 2010). Profit pursuit refers to firms cannot obtain sufficient returns by focusing on main business development will allocate a certain proportion of high-return financial assets to acquire a higher rate of return (Krippner, 2005). The third aspect if the factors influencing corporate financialization. Previous literature mainly examines the external environment, management characteristics, and other factors. Peng et al. (2018) pointed out that a higher-uncertainty environment decreases corporate financialization, Du et al. (2019) noted that a CEO’s financial background increases corporate financialization.

However, the existing literature provides no evidence on whether political turnover matters for corporate financialization, yet local policy preference and operating environment tend to change once an official transferred, which compels firms to adapt their investment strategies accordingly. Due to officials’ diverse preferences, abilities, and past experiences, political turnover will create uncertainty in policy execution, responsibility allocation and personnel transfer (Krueger and Walker, 2008; Xu and Wang, 2010). Moreover, the heterogeneity of officials will also lead to potential changes in future policies in the jurisdictions (Diebold and Yilmaz, 2009).

Moreover, entrepreneurship is a critical signal of economic development (Stamboulis and Barlas, 2014). Government policymakers will seek cultural, social, or financial benefits by entrepreneurial activities, and the entrepreneurship attitudes and skills can improve firms’ performance and promote economic development and employment (Bacigalupo et al., 2016).

### Research hypothesis

Previous research has shown that precautionary savings and profit pursuit are the primary motivations for holding financial assets. We analyze these motivations below.

1. Precautionary savings: Political turnover can increase corporate financialization through precautionary savings. In production and operation processes, future income, cost, and cash flow present many uncertainties. Internal cash and short-term financial assets will reduce the negative effect of capital chain rupture on production and operation activities (Opler et al., 1999). In addition, financial assets, as an investment opportunity, can be a hedging instrument when using options and derivatives, which can alleviate the negative effect of political turnover, reduce the uncertainty of future cash flows, and effectively disperse and hedge operational risks. In sum, firms can hold more financial assets to manage the impact of political turnover.
2. Profit pursuit: Political turnover can inhibit corporate financialization through profit pursuit from the following aspects. First, political turnover increases local business risks, risk premiums, and financing costs. The willingness of risk-averse managers to invest in financial assets will thus decrease (Luo et al., 2016). Second, the lending propensity of local banks will be affected by the official’s policy orientation, and the assessment of the actual loan risk and financing ability may be stricter and eventually reduce the loan scale. The phenomenon of a credit squeeze by banks will then limit corporate financing ability, intensify corporate financing constraints, and ultimately decrease the corporate financialization of NFCs (Baum et al., 2009). Financial assets that are held in pursuit of profit may therefore be affected by regulatory policy in the future and not be paid. Then, the asset price would suffer a sharp decline due to holders selling their assets, causing companies to reduce their holdings of financial assets. Therefore, we propose the following hypotheses:

*H1a*: Political turnover increases corporate financialization: the trend of corporate financialization increases.

*H1b*: Political turnover decreases corporate financialization: the trend of corporate financialization decreases.

Political turnover may affect corporate financialization through two channels: increasing physical investment and crowding-out operating cash. Firstly, due to China’s vertical administrative centralization, prefecture-city officials have substantial rights including allocate resources (Feng et al., 2019). Local government officials are absorbed to stimulate GDP growth from the view of political promotion tournaments (Zhou, 2004). Moreover, due to the limited tenure, promoted officials tend to intervene in the firms’ businesses to promote short-term GDP development (An et al., 2016). Further, local officials dominate local land supply and financial resources through state-owned banks. Specifically, to cater to the interests of local officials, enterprises prefer to overinvest in short-term physical projects that can stimulate local GDP to obtain critical resources, such as bank loans, tax preferences, and debt relief (Adhikari et al., 2006; Boubakri et al., 2008; Kim and Zhang, 2016). Thus, financial investments decrease accordingly. Therefore, we propose the following hypothesis.

*H2*: Political turnover reduces corporate financialization by increasing physical investment.

In addition, political connections are helpful for firms to obtain reliable information and government subsidies (Zhang et al., 2014). However, the expenses of constructing and sustaining political relations may deplete firms’ operating cash (Hill et al., 2014), thereby weakening their financial investment. Zhu and Zhang (2017) put forward that firms need to construct new political relations with local officials constantly when political turnover is high, which in turn hinders firm development. The bribery and efforts to construct networks with bureaucrats increase transaction costs and further limit the scope of operating cash flows (Xu et al., 2016). Companies also will increase the amount of donate money when city-level officials are replaced (Liu et al., 2021). In sum, firms may choose to sell their financial assets to release liquidity and reduce the uncertainty which caused by political turnover. Thus, we propose the following hypothesis.

*H3*: Political turnover reduces companies’ cash holdings and then decrease corporate financialization.

## Materials and methods

### Data and sample

Our data involves Chinese firms listed on the Shanghai and Shenzhen Stock Exchange’s A-share market from 2007 to 2020. We extract all firm-level data from the CSMAR databases. To clean the data, we (1) exclude financial firms, insurance and real-estate listed companies, and samples with missing data; (2) delete firms with less than 2 years’ available data in the database; (3) remove abnormal companies, such as “specially treated firms” and “particular transfer firms”; and (4) winsorize continuous variables at 1st and 99th percentiles to avoid extreme outlier effects.

We manually collect the personal information of the mayors and party secretaries. The data comes from people.com and xinhuanet.com. The details include the local officials’ ages and work experience. After gathering the data, we combine the data and obtain 995 mayors and 912 party secretaries’ information.

### Dependent variable

According to Demir (2009), and accounting standards, corporate financialization on stock *i* and year *t*, Fin*_i,t_*, is defined from broad and narrow perspectives. Broad financial assets (*Fin1*) are standardized by total assets and include monetary capital, trading financial assets, derivative financial assets, available-for-sale financial assets, held-to-maturity investments, long-term equity investments, dividend receivables, and interest receivables. In addition, narrow financial assets (*Fin2*) exclude long-term equity investments.

### Independent variable

Furthermore, refer to Li and Zhou (2005), we define *Turnover* variable. If the officials promote between January 1st and June 30th, we set the current year as the first year and *Turnover* equals1 and 0 otherwise. If the officials promote between July 1st and December 31st, then we set the following year as the first year and *Turnover* equals 1 and 0 otherwise.

### Empirical model

We use the following regression models to examine the impact of political turnover on corporate financialization. Fin*_i,t_* is the dependent variable and Turnover*_i,t_* is independent variables which has two alternatives. Turnover_*m* equals 1 for mayor’s turnover and 0 otherwise; Turnover_*s* equals 1 for party secretary’s turnover, and 0 otherwise. Moreover, Control*_i,t_* denotes a set of control variables that affect corporate financialization (see Table 1).

**Table 1:** Variable definitions.

Variables	Definition
*Fin1*	Broad financial asset holdings/total assets.
*Fin2*	Narrow financial asset holdings/total assets.
*Turnover_m*	The dummy variable equals 1 if the city where the firm’s register city is located experiences a mayor turnover and 0 otherwise.
*Turnover_s*	The dummy variable equals 1 if the city where the firm’s register city is located experiences a turnover of party secretary and 0 otherwise.
*Lev*	Total liability/total assets.
*Size*	Firm size, the natural logarithm of total assets.
*Age*	The natural logarithm of 1 + years of operation of the company from the registration of the firm to the end of the fiscal year.
*Soe*	A dummy variable, equals to 1 if the firm is a state-owned entity and equal to 0 otherwise.
*Roa*	Net profits/total assets.
*Growth*	Annual growth rate of total sales.
*Tq*	The ratio of the sum of market value of traded and non-traded shares and total debt to total assets.
*Gdp*	The growth rate of GDP


$\varepsilon_{i,t}$ is the error term.


(1) $$\begin{array}{c} {\mathrm{Fin}}_{i,t}=\alpha_{0}+\alpha_{1}{\mathrm{Turnover}}_{i,t}+\alpha_{k}{\mathrm{Control}}_{i,t}\\ +\mathrm{Year}+\mathrm{Industry}+\varepsilon \end{array}$$

According to Baron and Kenny (1986), the regression model designed to test H2 and H3 are listed below:


(2) $$\begin{array}{c} {\mathrm{Mediator}}_{i,t}=\beta_{0}+\beta_{1}{\mathrm{Turnover}}_{i,t}+\beta_{k}{\mathrm{Control}}_{i,t}\\ +\mathrm{Year}+\mathrm{Industry}+\varepsilon \end{array}$$

(3) $$\begin{array}{c} {\mathrm{Fin}}_{i,t}=\gamma_{0}+\gamma_{1}{\mathrm{Turnover}}_{i,t}+\gamma_{2}{\mathrm{Mediator}}_{i,t}\\ +\gamma_{k}{\mathrm{Control}}_{i,t}+\mathrm{Year}+\mathrm{Industry}+\varepsilon \end{array}$$

where Mediator represents the intermediary channels through which political turnover affects corporate financialization, including fixed asset investment (*fixed*) and cash holdings (*cash*).

We also control for the following variables: financial leverage (*Lev*), firm size (*Size*), firm age (*Age*), state ownership (*Soe*), return on assets (*Roa*), growth rate (*Growth*), Tobin’s Q (*Tq*), the growth rate of the province-level gross domestic product (*Gdp*), and year and industry dummy. Table 1 presents detailed definitions of these variables.

### Descriptive statistics

Table 2 reports the distribution of political turnover by year. The average of *Turnover* is 30.4% for mayors and 30.9% for party secretaries from 2007 to 2018. The three highest percentage of government official turnovers were in 2017, 2013 and 2018.

**Table 2:** Distribution of political turnover by year.

Year	Number of samples	Mayor turnover (%)	Party sectary turnover (%)
2007	1,124	0.310	0.335
2008	1,272	0.286	0.404
2009	1,361	0.138	0.0992
2010	1,673	0.183	0.184
2011	1941	0.299	0.230
2012	2095	0.282	0.270
2013	2053	0.418	0.431
2014	2037	0.127	0.151
2015	2,153	0.195	0.314
2016	2,532	0.275	0.236
2017	2,849	0.594	0.510
2018	3,141	0.342	0.385
Total	24,231	0.304	0.309

Table 3 presents the descriptive statistics of variables used in the regression. As shown, the average values of *Fin1* and *Fin2* are 0.25 and 0.216, respectively, and the standard deviations are 0.164 and 0.154, respectively. From the minimum and maximum of *Fin1* and *Fin2*, we can conclude that the trend of corporate financialization is polarized.

**Table 3:** Descriptive statistics.

Variable	Observation	Mean	SD	Min	Max
*Fin1*	24,231	0.25	0.164	0.0259	0.78
*Fin2*	24,231	0.216	0.154	0.0151	0.734
*Turnoverm*	24,231	0.304	0.46	0	1
*Turnovers*	24,231	0.309	0.462	0	1
*Lev*	24,231	0.422	0.212	0.0474	0.981
*Size*	24,231	21.92	1.251	19.49	25.81
*Age*	24,231	1.919	0.915	0	3.219
*Soe*	24,231	0.401	0.49	0	1
*Roa*	24,231	0.039	0.0625	−0.279	0.2
*Growth*	24,231	0.174	0.398	−0.561	2.592
*Tq*	24,231	2.078	1.315	0.907	8.804
*Gdp*	24,231	0.0924	0.0259	0.0364	0.16

All variables are defined in Table 1.

Table 4 summarize the Pearson and Spearman correlations for key variables. The results shows that Tunover_m and Turnover_s are significantly and negatively correlated with corporate financialization.

**Table 4:** Summary statistics and correlation.

	*Fin1*	*Fin2*	*Turnover_m*	*Turnover_s*	*Lev*	*Size*	*Age*	*Soe*	*Roa*	*Growth*	*Tq*	*Gdp*
*Fin1*	1											
*Fin2*	0.90***	1										
*Turnover_m*	0.04***	0.03***	1									
*Turnover_s*	0.03***	0.03***	0.30***	1								
*Lev*	0.40***	0.42***	−0.02***	−0.01*	1							
*Size*	0.17***	0.22***	0.00	0.00	0.43***	1						
*Age*	0.19***	0.31***	−0.01**	−0.00	0.40***	0.36***	1					
*Soe*	0.08***	0.15***	−0.04***	−0.04***	0.31***	0.34***	0.43***	1				
*Roa*	0.23***	0.25***	−0.00	−0.01	0.40***	0.01	0.25***	0.10***	1			
*Growth*	0.05***	0.03***	0.01**	0.01	0.04***	0.05***	0.01**	0.05***	0.21***	1		
*Tq*	0.11***	0.10***	−0.04***	−0.03***	0.18***	0.39***	0.08***	0.12***	0.06***	0.01**	1	
*Gdp*	0.05***	0.03***	−0.08***	−0.06***	0.10***	0.15***	0.04***	0.20***	0.03***	0.03***	−0.01*	1

Lower-triangular cells report Pearson’s correlation coefficients, upper-triangular cells are Spearman’s rank correlation, ^***^p < 0.01, ^**^p < 0.05, ^*^p < 0.1.

## Results

### Baseline results

Table 5 shows the baseline results. To test whether political turnover affects corporate financialization, we divide political turnover in three groups: mayor’s turnover, party secretary’s turnover and turnover of both mayor and party secretary. From Table 5, the coefficient of *Turnover-m* in the change for mayor only group (columns 1–2) and the coefficient of *Turnover_both* in change for both mayor and party secretary group (columns 5–6) show significantly negative effect on corporate financialization, while the coefficient of *Turnover_ s* in change of party secretary only group (columns 3–4) has no significant influence. Columns (1), (3) and (5) use *Fin1* as the dependent variable, and columns (2), (4) and (6) show the results by using *Fin2*. The reason may by that mayor is actually the chief administrator who constructs and executes local social and economic policies, while party secretary is appointed to supervise the local government. Moreover, Zheng et al. (2014) reports that a mayor has a closer relationship with the company. Overall, the empirical results confirm H1b.

**Table 5:** Baseline findings.

	(1)	(2)	(3)	(4)	(5)	(6)
	*Fin1*	*Fin2*	*Fin1*	*Fin2*	*Fin1*	*Fin2*
*Turnover_m*	−0.0058^***^	−0.0048^***^				
	(0.0016)	(0.0015)				
*Turnover_s*			−0.0003	−0.0019		
			(0.0016)	(0.0015)		
*Turnover_both*					−0.0066^***^	−0.0057^***^
					(0.0022)	(0.0020)
*Lev*	−0.2984^***^	−0.2448^***^	−0.2984^***^	−0.2448^***^	−0.2985^***^	−0.2449^***^
	(0.0144)	(0.0121)	(0.0144)	(0.0121)	(0.0144)	(0.0121)
*Size*	0.0074^***^	0.0035^*^	0.0075^***^	0.0035^*^	0.0075^***^	0.0035^*^
	(0.0024)	(0.0021)	(0.0024)	(0.0021)	(0.0024)	(0.0021)
*Age*	−0.0079^***^	−0.0274^***^	−0.0078^***^	−0.0273^***^	−0.0079^***^	−0.0274^***^
	(0.0028)	(0.0023)	(0.0028)	(0.0023)	(0.0028)	(0.0023)
*SOE*	0.0071	0.0095^**^	0.0071	0.0095^**^	0.0071	0.0095^**^
	(0.0052)	(0.0044)	(0.0053)	(0.0044)	(0.0053)	(0.0044)
*Roa*	0.1558^***^	0.1727^***^	0.1561^***^	0.1729^***^	0.1557^***^	0.1726^***^
	(0.0305)	(0.0273)	(0.0305)	(0.0274)	(0.0305)	(0.0274)
*Growth*	−0.0251^***^	−0.0146^***^	−0.0250^***^	−0.0146^***^	−0.0250^***^	−0.0145^***^
	(0.0028)	(0.0025)	(0.0028)	(0.0025)	(0.0028)	(0.0025)
*Tobin’s Q*	0.0079^***^	0.0058^***^	0.0079^***^	0.0059^***^	0.0079^***^	0.0058^***^
	(0.0018)	(0.0017)	(0.0018)	(0.0017)	(0.0018)	(0.0017)
*Gdp*	−0.4732^***^	−0.3525^***^	−0.4705^***^	−0.3490^***^	−0.4728^***^	−0.3522^***^
	(0.1214)	(0.1054)	(0.1217)	(0.1056)	(0.1214)	(0.1054)
*Constant*	0.2516^***^	0.2899^***^	0.2492^***^	0.2882^***^	0.2497^***^	0.2884^***^
	(0.0531)	(0.0470)	(0.0531)	(0.0470)	(0.0531)	(0.0470)
*N*	24,231	24,231	24,231	24,231	24,231	24,231
*R* ^2^	0.2630	0.2915	0.2628	0.2913	0.2630	0.2915
Year	Yes	Yes	Yes	Yes	Yes	Yes
Ind	Yes	Yes	Yes	Yes	Yes	Yes

^*^, ^**^ and ^***^ indicate significance at the 10, 5 and 1% levels.

### Robustness tests

#### Potential endogenous problems

Our results may be affected by missing variables, reverse causality, and sample selection bias. Although we include some control variables to reduce endogenous problems, corporate financialization can also be affected by some unobservable factors. Therefore, we use two-stage least squares (2SLS) and propensity score matching (PSM) methods to address endogeneity issues.

First, in the 2SLS approach, we follow Deng et al. (2019) in choosing “workplace connection” (*Workplace*) as the instrumental variable for political turnover. Table 6 shows the results. The coefficient of *Workplace* in column (1) is significantly negative. The relative test value is greater than 10, indicating no insufficient identification or weak IV problems. Columns (2) and (3) show the results of second-stage regression. The coefficients of *Pre_turnover_m* are negative and statistically significant, indicating that political turnover inhibits corporate financialization which is consistent with Table 5.

**Table 6:** Dealing with the potential endogenous problem.

	2SLS regression			PSM	
	1st stage	2nd stage	2nd stage	2nd stage	2nd stage
	*Turnover_m*	*Fin1*	*Fin2*	*Fin1*	*Fin2*
	(1)	(2)	(3)	(4)	(5)
*Workplace*	−0.0389^***^				
	(0.0059)				
*Pre_turnover_m*		−0.5678^***^	−0.4158^***^		
		(0.0971)	(0.0757)		
*Turnover_m*				−0.0058^***^	−0.0048^**^
				(0.0020)	(0.0019)
*Lev*	−0.0032	−0.2989^***^	−0.2452^***^	−0.2984^***^	−0.2448^***^
	(0.0179)	(0.0120)	(0.0095)	(0.0069)	(0.0062)
*Size*	−0.0017	0.0062^***^	0.0026	0.0074^***^	0.0035^***^
	(0.0032)	(0.0021)	(0.0016)	(0.0011)	(0.0010)
*Age*	−0.0012	−0.0085^***^	−0.0278^***^	−0.0079^***^	−0.0274^***^
	(0.0040)	(0.0026)	(0.0021)	(0.0014)	(0.0013)
*Soe*	−0.0121^*^	−0.0006	0.0039	0.0071^***^	0.0095^***^
	(0.0072)	(0.0049)	(0.0038)	(0.0023)	(0.0021)
*Roa*	−0.0698	0.1214^***^	0.1475^***^	0.1558^***^	0.1727^***^
	(0.0550)	(0.0368)	(0.0287)	(0.0202)	(0.0178)
*Growth*	−0.0043	−0.0273^***^	−0.0162^***^	−0.0251^***^	−0.0146^***^
	(0.0075)	(0.0050)	(0.0039)	(0.0027)	(0.0024)
*Tq*	−0.0003	0.0074^***^	0.0054^***^	0.0079^***^	0.0058^***^
	(0.0027)	(0.0018)	(0.0015)	(0.0011)	(0.0010)
*Gdp*	−0.6036^***^	−0.7013^***^	−0.5193^***^	−0.4732^***^	−0.3525^***^
	(0.1951)	(0.1334)	(0.1046)	(0.0669)	(0.0600)
*Constant*	0.4565^***^	0.4899^***^	0.4643^***^	0.2516^***^	0.2899^***^
	(0.0768)	(0.0648)	(0.0507)	(0.0251)	(0.0230)
*N*	24,231	24,231	24,231	24,231	24,231
*R* ^2^	0.0866	−2.0109	−1.0918	0.2630	0.2915
Year	Yes	Yes	Yes	Yes	Yes
Ind	Yes	Yes	Yes	Yes	Yes

^*^, ^**^ and ^***^ indicate significance at the 10, 5 and 1% levels.

Second, we use the PSM model to create a control sample without political turnover and a treatment sample that experiences political turnover, which can capture differences between treatment and control firms. Specifically, we calculate a propensity score for each firm-year observation by running a Probit model, which regresses *Trunover_m* on county economic indicators and firm factors, involving *per capita* GDP (*Pgdp*), *Lev*, *Size*, *Soe*, *Roa*, *Growth* and *Tq*, *Industry*, and *Year* dummy. Moreover, each company in the treatment group (experiencing political turnover) matches the control company (not experiencing political turnover) with the nearest neighbor matching method. After matching, we conduct a second-stage regression to analyze how political turnover affects corporate financialization. Columns (4) and (5) in Table 6 show the estimated results of *Turnover_m* is still negative and significant, which is still consistent with the baseline results.

#### Alternative measures of political turnover

First, referring to Wang and Xu (2008), we change the measurement of *Turnover_m*. Specifically, we set the current year as their first year when the mayor take office, and *Turnover_m2* equals 1 and 0 otherwise. The specific month of *Turnover* is ignored. Columns (1) and (2) in Table 7 show that political turnover still reduces corporate financialization.

**Table 7:** Alternative measures of political turnover.

	*Fin1*	*Fin2*	Fin1	Fin2
	(1)	(2)	(3)	(4)
*Turnover_m2*	−0.0063^***^	−0.0048^**^		
	(0.0021)	(0.0019)		
*Turnover_m*			−0.0054***	−0.0043**
			(0.0021)	(0.0019)
*Lev*	−0.2983^***^	−0.2448^***^	−0.3033***	−0.2473***
	(0.0069)	(0.0062)	(0.0071)	(0.0064)
*Size*	0.0074^***^	0.0035^***^	0.0074***	0.0038***
	(0.0011)	(0.0010)	(0.0011)	(0.0010)
*Age*	−0.0079^***^	−0.0274^***^	−0.0072***	−0.0273***
	(0.0014)	(0.0013)	(0.0014)	(0.0013)
*Soe*	0.0071^***^	0.0095^***^	0.0059**	0.0091***
	(0.0023)	(0.0021)	(0.0024)	(0.0021)
*Roa*	0.1556^***^	0.1727^***^	0.1566***	0.1688***
	(0.0202)	(0.0178)	(0.0206)	(0.0182)
*Growth*	−0.0251^***^	−0.0146^***^	−0.0250***	−0.0149***
	(0.0027)	(0.0024)	(0.0028)	(0.0025)
*Tq*	0.0079^***^	0.0058^***^	0.0072***	0.0056***
	(0.0011)	(0.0010)	(0.0011)	(0.0010)
*Gdp*	−0.4739^***^	−0.3525^***^	−0.6027***	−0.4261***
	(0.0669)	(0.0600)	(0.0719)	(0.0645)
*Constant*	0.2513^***^	0.2899^***^	0.2753***	0.2970***
	(0.0251)	(0.0230)	(0.0261)	(0.0241)
*N*	24,911	24,911	23,372	23,372
*R* ^2^	0.2527	0.2845	0.2578	0.2858
Year	Yes	Yes	Yes	Yes
Ind	Yes	Yes	Yes	Yes

^*^, ^**^ and ^***^ indicate significance at the 10, 5 and 1% levels.

Moreover, promotional opportunities may drive local officials to influence the real economy. We eliminate the sample of officials who lose promotion opportunities because of the promotion age limit. As the promotion age of vice-ministerial officials and department-level cadres is limited to 58 years and that of ministerial leaders is 67 years, we remove officials aged over 58 years in prefecture-level cities and those aged over 67 years in Beijing and Shanghai. The results are shown in columns (5) and (6) of Table 7, which is consistent with Table 5.

#### The influence of the party congress

The 17th, 18th, and 19th Congress of the Communist Party of China were held in 2007, 2012, and 2017, respectively, which may have impacted our baseline results. Thus, we excluded samples from 2007, 2012, and 2017. The results are shown in Table 8.

**Table 8:** The influence of the Party Congress.

	*Fin1*	*Fin2*
	(1)	(2)
*Turnover_m*	−0.0075^***^	−0.0055^**^
	(0.0024)	(0.0022)
*Lev*	−0.2975^***^	−0.2446^***^
	(0.0080)	(0.0070)
*Size*	0.0072^***^	0.0033^***^
	(0.0012)	(0.0011)
*Age*	−0.0103^***^	−0.0303^***^
	(0.0016)	(0.0015)
*Soe*	0.0080^***^	0.0112^***^
	(0.0027)	(0.0024)
*Roa*	0.1437^***^	0.1606^***^
	(0.0225)	(0.0195)
*Growth*	−0.0275^***^	−0.0174^***^
	(0.0032)	(0.0029)
*Tq*	0.0076^***^	0.0056^***^
	(0.0012)	(0.0011)
*Gdp*	−0.4825^***^	−0.3464^***^
	(0.0760)	(0.0677)
*Constant*	0.2432^***^	0.2848^***^
	(0.0278)	(0.0254)
*N*	18,669	18,669
*R* ^2^	0.2585	0.2928
Year	Yes	Yes
Ind	Yes	Yes

^*^, ^**^ and ^***^ indicate significance at the 10, 5 and 1% levels.

## Heterogeneity of official and companies

### External appointment and local promotion

Persson and Zhuravskaya (2016) pointed out that the relationship between governments and enterprises can be expressed by whether the mayor is promoted locally. Officials promoted within their prefecture city typically already have established networks with local firms. The support of local companies is a contributing factor for the political promotion. However, this support will constrain the mayors’ abilities to perform their duties. Conversely, officials appointed from other prefecture-cities will not have the constraints (Shleifer and Summers, 2013). Moreover, different promotion methods mean different experiences and abilities and represent significant differences in political incentives (Wang and Xu, 2008). Therefore, we further characterize the types of turnover: whether the new official is external appointment or local promotion.

Table 9 show the empirical results. The coefficients of *Turnover_m* are significantly negative when the new official is externally appointed.

**Table 9:** External appointment and local promotion.

	*Fin1*		*Fin2*	
	External	Local	External	Local
	(1)	(2)	(3)	(4)
*Turnover_m*	−0.0071^**^	−0.0039	−0.0058^**^	−0.0034
	(0.0032)	(0.0026)	(0.0029)	(0.0024)
*Lev*	−0.3249^***^	−0.2693^***^	−0.2679^***^	−0.2197^***^
	(0.0101)	(0.0094)	(0.0089)	(0.0085)
*Size*	0.0073^***^	0.0073^***^	0.0025^*^	0.0041^***^
	(0.0015)	(0.0015)	(0.0013)	(0.0013)
*Age*	−0.0082^***^	−0.0082^***^	−0.0305^***^	−0.0243^***^
	(0.0019)	(0.0020)	(0.0018)	(0.0018)
*Soe*	0.0049	0.0070^**^	0.0130^***^	0.0038
	(0.0034)	(0.0032)	(0.0030)	(0.0028)
*Roa*	0.1559^***^	0.1648^***^	0.1830^***^	0.1681^***^
	(0.0298)	(0.0276)	(0.0255)	(0.0250)
*Growth*	−0.0266^***^	−0.0237^***^	−0.0160^***^	−0.0137^***^
	(0.0039)	(0.0037)	(0.0034)	(0.0034)
*Tobin Q*	0.0074^***^	0.0079^***^	0.0042^***^	0.0073^***^
	(0.0014)	(0.0015)	(0.0013)	(0.0015)
*Gdp*	−0.4792^***^	−0.1677	−0.3980^***^	−0.0595
	(0.0879)	(0.1037)	(0.0789)	(0.0932)
*Constant*	0.2791^***^	0.1865^***^	0.3325^***^	0.2159^***^
	(0.0358)	(0.0351)	(0.0335)	(0.0317)
*N*	12,050	12,181	12,050	12,181
*R* ^2^	0.2823	0.2395	0.3172	0.2648
Year	Yes	Yes	Yes	Yes
Ind	Yes	Yes	Yes	Yes

^*^, ^**^ and ^***^ indicate significance at the 10, 5 and 1% levels.

### The age of new officials

The influence and motivation of officials with different characteristics are quite different (Qian and Xu, 2014). In the evaluation of official promotions, age is a very important factor that affects the incentive for political promotion. Under the compulsory retirement system, the promotion possibility of prefecture-level city officials gradually decreases after the age of 56. Therefore, we take the age of 56 years as the standard for analysis. Table 10 demonstrate that the effect is more pronounced when the official is under 56 years old. The age advantage intensifies the impulse of local government officials to reduce corporate financialization. In contrast, when the officials are older, the future promotion space is limited, and the reduction in promotion expectations affects the role of the promotion incentive.

**Table 10:** The age of new officials.

	*Fin1*		*Fin2*	
	Above 56	Below 56	Above 56	Below 56
	(1)	(2)	(3)	(4)
*Turnover_m*	0.0067	−0.0044^*^	0.0058	−0.0035^*^
	(0.0057)	(0.0022)	(0.0052)	(0.0021)
*Lev*	−0.3258^***^	−0.2877^***^	−0.2869^***^	−0.2293^***^
	(0.0136)	(0.0080)	(0.0126)	(0.0071)
*Size*	0.0045^**^	0.0089^***^	−0.0011	0.0060^***^
	(0.0019)	(0.0013)	(0.0017)	(0.0012)
*Age*	−0.0049^*^	−0.0094^***^	−0.0233^***^	−0.0295^***^
	(0.0025)	(0.0016)	(0.0023)	(0.0015)
*Soe*	0.0137^***^	0.0025	0.0130^***^	0.0064^***^
	(0.0048)	(0.0026)	(0.0043)	(0.0023)
*Roa*	0.1844^***^	0.1400^***^	0.1979^***^	0.1573^***^
	(0.0429)	(0.0228)	(0.0380)	(0.0201)
*Growth*	−0.0317^***^	−0.0227^***^	−0.0170^***^	−0.0140^***^
	(0.0051)	(0.0032)	(0.0048)	(0.0028)
*Tobin Q*	0.0081^***^	0.0078^***^	0.0050^***^	0.0063^***^
	(0.0021)	(0.0012)	(0.0019)	(0.0011)
*Gdp*	−0.3062^***^	−0.3853^***^	−0.2823^***^	−0.2518^***^
	(0.1110)	(0.0860)	(0.1027)	(0.0756)
*Constant*	0.3082^***^	0.2083^***^	0.4132^***^	0.2176^***^
	(0.0492)	(0.0298)	(0.0453)	(0.0274)
*N*	6,768	17,463	6,768	17,463
*R* ^2^	0.2917	0.2473	0.3285	0.2735
Year	Yes	Yes	Yes	Yes
Ind	Yes	Yes	Yes	Yes

^*^, ^**^ and ^***^ indicate significance at the 10, 5 and 1% levels.

### Ownership structure

Concentrated ownership is a unique feature of Chinese firms, which means that the government is the biggest shareholder (Sun and Tong, 2003). State-owned enterprises’ directors are usually assigned by government and carry bureaucratic ranks. Their objectives are to carry out policies, and not to maximize shareholders’ profits. Therefore, we construct a dummy variable, *Soe*, which denoting whether a company is controlled by the government. Table 11 shows that the coefficient of *Soe* is not significant, while the coefficient of *non-Soe* is significant, which suggests non-SOEs decrease corporate financialization further when prefecture-city officials’ turnover. SOEs are generally large-scale and deep-rooted enterprises that are widely distributed and less affected by political changes in a single region, while non-SOEs are more dependent on local policies. Therefore, local political turnover has a more significant effect on non-SOEs’ financialization. Our result is consistent with the conclusion of (Wang and Wen, 2019).

**Table 11:** Ownership structure.

	*Fin1*		*Fin2*	
	Soe	Non-Soe	Soe	Non-Soe
	(1)	(2)	(3)	(4)
*Turnover_m*	−0.0042	−0.0067^**^	−0.0024	−0.0064^**^
	(0.0031)	(0.0026)	(0.0027)	(0.0025)
*Lev*	−0.2760^***^	−0.3109^***^	−0.1902^***^	−0.2758^***^
	(0.0107)	(0.0090)	(0.0089)	(0.0084)
*Size*	0.0048^***^	0.0144^***^	0.0009	0.0095^***^
	(0.0014)	(0.0017)	(0.0012)	(0.0016)
*Age*	0.0048^**^	−0.0161^***^	−0.0179^***^	−0.0329^***^
	(0.0024)	(0.0018)	(0.0022)	(0.0017)
*Soe*	0.0000	0.0000	0.0000	0.0000
	(.)	(.)	(.)	(.)
*Roa*	0.2328^***^	0.0987^***^	0.2702^***^	0.1125^***^
	(0.0336)	(0.0255)	(0.0265)	(0.0236)
*Growth*	−0.0157^***^	−0.0270^***^	−0.0009	−0.0191^***^
	(0.0039)	(0.0036)	(0.0035)	(0.0033)
*Tobin Q*	0.0079^***^	0.0116^***^	0.0080^***^	0.0085^***^
	(0.0017)	(0.0013)	(0.0016)	(0.0013)
*Gdp*	−0.3886^***^	−0.3561^***^	−0.1894^**^	−0.3468^***^
	(0.0865)	(0.1027)	(0.0741)	(0.0941)
*Constant*	0.2670^***^	0.1037^***^	0.2844^***^	0.1838^***^
	(0.0350)	(0.0388)	(0.0319)	(0.0360)
*N*	9,714	14,517	9,714	14,517
*R* ^2^	0.2473	0.2998	0.2538	0.3283
Year	Yes	Yes	Yes	Yes
Ind	Yes	Yes	Yes	Yes

^*^, ^**^ and ^***^ indicate significance at the 10, 5 and 1% levels.

### Firm size

Small-sized firms tend to have more agency problems, make inefficient investment decisions, and face higher operating risks (Ho and Wong, 2001). Large firms always have more capital assets, mature investment portfolios, and anti-risk abilities. We expect that the management of small-sized firms may have a greater incentive to pursue financial investment to cover up the negative influences. We split our data into two clusters based on the firm size’s median. Table 12 show that the coefficient of large-sized companies is negative but not significant, while the coefficient of small-sized companies is significantly negative. Official’s turnover has a greater significant impact on the corporate financialization in small-sized firms.

**Table 12:** Firm size.

	*Fin1*		*Fin2*	
	Large size	Small size	Large size	Small size
	(1)	(2)	(3)	(4)
*Turnover_m*	−0.0042	−0.0068^**^	−0.0030	−0.0059^***^
	(0.0028)	(0.0028)	(0.0025)	(0.0027)
*Lev*	−0.2634^***^	−0.3240^***^	−0.1838^***^	−0.2851^***^
	(0.0106)	(0.0090)	(0.0093)	(0.0082)
*Size*	0.0102^***^	−0.0027	0.0053^***^	−0.0056^**^
	(0.0017)	(0.0028)	(0.0015)	(0.0027)
*Age*	−0.0004	−0.0130^***^	−0.0207^***^	−0.0314^***^
	(0.0022)	(0.0019)	(0.0020)	(0.0018)
*Soe*	0.0060^*^	0.0097^***^	0.0093^***^	0.0108^***^
	(0.0031)	(0.0035)	(0.0027)	(0.0032)
*Roa*	0.1891^***^	0.1302^***^	0.2210^***^	0.1386^***^
	(0.0307)	(0.0271)	(0.0254)	(0.0244)
*Growth*	−0.0178^***^	−0.0322^***^	−0.0089^***^	−0.0201^***^
	(0.0034)	(0.0041)	(0.0031)	(0.0037)
*Tobin Q*	0.0091^***^	0.0091^***^	0.0095^***^	0.0065^***^
	(0.0019)	(0.0013)	(0.0017)	(0.0012)
*Gdp*	−0.4776^***^	−0.4985^***^	−0.3086^***^	−0.4381^***^
	(0.0923)	(0.0957)	(0.0802)	(0.0869)
*Constant*	0.1633^***^	0.4772^***^	0.1990^***^	0.5078^***^
	(0.0403)	(0.0607)	(0.0359)	(0.0571)
*N*	10,963	13,268	10,963	13,268
*R* ^2^	0.2277	0.2918	0.2363	0.3226
Year	Yes	Yes	Yes	Yes
Ind	Yes	Yes	Yes	Yes

^*^, ^**^ and ^***^ indicate significance at the 10, 5 and 1% levels.

## Mediating effect

### Fixed asset investment

Under the continuous prosperity of the capital market, the rate of return on financial investment remains high. Firms are more willing to invest in financial assets than fixed asset investment with more significant uncertainty and longer return. Moreover. financial investments will occupy the resources originally used for fixed asset investment. Existing literature shows that political turnover increases a firm’s fixed asset investment in its jurisdiction (Chen and Luo, 2012); that is, political turnover leads firms investing their resources in fixed asset investment, thus inhibiting corporate financial investment. Therefore, we introduce fixed asset investment as an intermediary variable to test H2. Referring to Du et al. (2017), we define fixed asset investment (Fixed) as:


$$\mathrm{Fixed}=\cdot \left(\begin{array}{l} \mathrm{Fixed assets}+\mathrm{Construction in process}\\ +\mathrm{Engineer material} \end{array}\right)/\mathrm{Total asset}.$$

Table 13 columns (1)–(3) reports the mediating effects of fixed asset investment. The coefficient is 0.0046 and significant, showing that political turnover increases fixed asset investment. Columns (2) and (3) report the coefficients of the explanatory and mediatory variables, which are significant. Thus, fixed asset investment plays a partially mediating role.

**Table 13:** Mediating effect and Sobel test.

	*Fixed*	*Fin1*	*Fin2*	*Cash*	*Fin1*	*Fin2*
	(1)	(2)	(3)	(4)	(5)	(6)
*Turnover_m*	0.0046^**^	−0.0041^**^	−0.0034^*^	−0.0039**	−0.0025*	−0.0012
	(0.0023)	(0.0018)	(0.0017)	(0.0017)	(0.0014)	(0.0010)
*Fixed*		−0.3793^***^	−0.3030^***^			
		(0.0052)	(0.0047)			
*Cash*					0.8507***	0.9125***
					(0.0056)	(0.0046)
*Lev*	0.0838^***^	−0.2666^***^	−0.2194^***^	−0.2359***	−0.0992***	−0.0308***
	(0.0070)	(0.0063)	(0.0058)	(0.0055)	(0.0058)	(0.0041)
*Size*	0.0094^***^	0.0110^***^	0.0063^***^	−0.0001	0.0077***	0.0038***
	(0.0013)	(0.0009)	(0.0009)	(0.0009)	(0.0007)	(0.0005)
*Age*	0.0065^***^	−0.0054^***^	−0.0254^***^	−0.0352***	0.0222***	0.0049***
	(0.0014)	(0.0012)	(0.0012)	(0.0012)	(0.0010)	(0.0007)
*Soe*	0.0373^***^	0.0212^***^	0.0208^***^	0.0220***	−0.0117***	−0.0106***
	(0.0027)	(0.0021)	(0.0019)	(0.0018)	(0.0017)	(0.0012)
*Roa*	−0.2027^***^	0.0789^***^	0.1113^***^	0.2197***	−0.0237	−0.0216**
	(0.0209)	(0.0187)	(0.0169)	(0.0158)	(0.0151)	(0.0101)
*Growth*	−0.0190^***^	−0.0323^***^	−0.0203^***^	−0.0119***	−0.0163***	−0.0047***
	(0.0029)	(0.0025)	(0.0023)	(0.0022)	(0.0019)	(0.0013)
*Tobin Q*	−0.0079^***^	0.0049^***^	0.0034^***^	0.0069***	0.0019***	−0.0005
	(0.0010)	(0.0010)	(0.0009)	(0.0009)	(0.0007)	(0.0005)
*Gdp*	0.6164^***^	−0.2394^***^	−0.1657^***^	−0.3119***	−0.2109***	−0.0695**
	(0.0782)	(0.0598)	(0.0553)	(0.0559)	(0.0492)	(0.0331)
*Constant*	0.0179	0.2584^***^	0.2954^***^	0.3367***	−0.0363**	−0.0190*
	(0.0300)	(0.0224)	(0.0211)	(0.0212)	(0.0171)	(0.0116)
*N*	24,231	24,231	24,231	24,231	24,231	24,231
*R* ^2^	0.3086	0.3967	0.3884	0.3449	0.6508	0.7982
Year	Yes	Yes	Yes	Yes	Yes	Yes
Ind	Yes	Yes	Yes	Yes	Yes	Yes

^*^, ^**^ and ^***^ indicate significance at the 10, 5 and 1% levels.

### Cash holdings

The expenses of reconstructing and continuing political relations may be costly and reduce firms’ internal cash. Moreover, political turnover will cause firms to lose their original government subsidies, and firms may have to carry out additional rent-seeking activities. Therefore, we introduce the level of cash holdings as an intermediary variable to test Hypothesis 3. We define cash holdings (*Cash*) as:


Cash=Cash and cash equivalents attheendof the period/Total assets

Table 13 columns (4)–(6) reports the mediating effects of corporate cash holdings. The coefficient in column (4) is −0.0039 and significant. Columns (5) and (6) show that the coefficient of the mediating variable is positive and significant, while the coefficient of the explanatory variable is not significant. Hence, corporate cash holdings have a perfect mediating role.

## Conclusion and implications

### Conclusion

With the deepening of reform and opening up of the China economy, the internal and external environments of the Chinese capital market have undergone tremendous changes. There are many uncertainties surrounding political turnover and corporate financialization. This study examines the relationship between political turnover and corporate financialization. (1) We discover that the mayor turnover significantly decreases corporate financialization, especially when the mayor is promoted into that position from another region and ages below 56 years. (2) Our heterogeneity analysis reveals that the correlation between political turnover and corporate financialization is more significant in non-SOEs and small-sized firms. (3) We also investigate the mechanism by which political turnover influences corporate financialization. The results show that fixed asset investment and cash holdings have significant mediating effects. (4) The robustness test is consistent with the baseline regression after considering endogeneity and measurement errors. The results indicate that the uncertainties brought about by political turnover have a significant influence on corporate financialization in the officials’ jurisdictions, and this effect varies with official and firm characteristics.

### Implications

1. The change in the institutional environment formed by political turnover has an inhibitory effect on corporate financialization. When considering political promotions, especially for mayors (the chief officials) of prefectures, the superior government should pay attention to the effect of such appointments on GDP development and the financial implications for the companies within the relevant jurisdictions. From the perspective of “from virtue to real,” the official’s promotion needs to consider their professional abilities, work experience, and personal characteristics such as cadres exchange in a new spot, age, and educational background, especially in the eastern regions. In addition, institutional environmental differences and credit discrimination should be eliminated. Moreover, the financial market should support the real economy and increase the efficiency of capital allocation. Capital market and financial assets should be further developed so that firms can obtain a higher return from the real economy. Furthermore, companies can adopt digital transition to make investment decisions to better adapt to political turnover and other changes in the external environment. Finally, managers need to explore co-creation with government officials to obtain key resources, such as bank loans, tax incentives and debt relief, to seek better opportunities.
2. We should realize that corporate behavior is complex, and there are various internal reasons for corporate financial investments. This paper analyses how to inhibit corporate financialization from the perspective of official turnover. Moreover, the data we used may have some limitations. Considering the availability of corporate financial data, this paper selects listed companies as the research sample, and does not involve non-listed companies. Although these companies have some representatives, the influence of sample selection cannot be completely excluded. Finally, this paper is mainly based on the existing theories and uses the qualitative analysis method to infer research hypotheses. There is a lack of scientific mathematical derivation of the internal mechanism of official turnover affecting corporate financialization, and the inference process is inevitably constrained by subjective experience. Therefore, the derivation of research hypotheses in this paper contains the author’s subjective tendency, so other important issues to be studied may be omitted.
3. This paper uses empirical research methods to explore the impact of official turnover on corporate financialization. Subsequent research can try to obtain first-hand information through field research and case analysis methods, to visually reveal a series of performance of a single enterprise in official turnover, so as to better understand the various countermeasures that enterprises may take when faced with changes in the policy environment caused by official turnover. Finally, relevant research directions in the future can start from green practices and digital technologies.

## Data availability statement

The raw data supporting the conclusions of this article will be made available by the authors, without undue reservation.

## Author contributions

SL: conceptualization and Writing - original draft. YQ: methodology and Writing - review and editing. SY: data curation. SD: software. All authors contributed to the article and approved the submitted version.

## Funding

This work was supported by National Natural Science Foundation of China (No. 71873027) and Humanities and Social Science Foundation of Ministry of Education of China (18YJA790063).

## Conflict of interest

The authors declare that the research was conducted in the absence of any commercial or financial relationships that could be construed as a potential conflict of interest.

## Publisher’s note

All claims expressed in this article are solely those of the authors and do not necessarily represent those of their affiliated organizations, or those of the publisher, the editors and the reviewers. Any product that may be evaluated in this article, or claim that may be made by its manufacturer, is not guaranteed or endorsed by the publisher.

## References

[ref1] Adhikari A. Derashid C. Zhang H. (2006). Public policy, political connections, and effective tax rates: longitudinal evidence from Malaysia. J. Account. Public Policy 25, 574–595. doi: 10.1016/j.jaccpubpol.2006.07.001

[ref2] An H. Chen Y. Luo D. Zhang T. (2016). Political uncertainty and corporate investment: evidence from China. J. Corp. Finan. 36, 174–189. doi: 10.1016/j.jcorpfin.2015.11.003

[ref001] Bacigalupo M. Kampylis P. Punie Y. Van den Brande G. (2016). EntreComp: the entrepreneurship competence framework. Luxembourg: Publication Office of the European Union.

[ref002] Baron R. M. Kenny D. A. (1986). The moderator-mediator variable distinction in social psychological research: conceptual, strategic, and statistical considerations. J. Pers. Soc. Psychol. 51, 1173–1182. doi: 10.1037/0022-3514.51.6.1173 3806354

[ref3] Barradas R. Lagoa S. (2017). Financialization and Portuguese real investment: a supportive or disruptive relationship? J. Post Keynes. Econ. 40, 413–439. doi: 10.1080/01603477.2017.1286940

[ref4] Baum C. F. Caglayan M. Ozkan N. (2009). The second moments matter: the impact of macroeconomic uncertainty on the allocation of loanable funds. Econ. Lett. 102, 87–89. doi: 10.1016/j.econlet.2008.11.019

[ref5] Bhattacharya U. Hsu P.-H. Tian X. Xu Y. (2017). What affects innovation more: policy or policy uncertainty? J. Financ. Quant. Anal. 52, 1869–1901. doi: 10.1017/S0022109017000540

[ref6] Boubakri N. Cosset J.-C. Saffar W. (2008). Political connections of newly privatized firms. J. Corp. Finan. 14, 654–673. doi: 10.1016/j.jcorpfin.2008.08.003

[ref7] Chen Y. Luo D. (2012). Local Governors' turnover and Firms' Investment. Econ. Res. J. 47, 18–30.

[ref8] Demir F. (2009). Financial liberalization, private investment and portfolio choice: Financialization of real sectors in emerging markets. J. Dev. Econ. 88, 314–324. doi: 10.1016/j.jdeveco.2008.04.002

[ref9] Deng Y. Wu Y. Xu H. (2019). Political turnover and firm pollution discharges: An empirical study. China Econ. Rev. 58:101363. doi: 10.1016/j.chieco.2019.101363

[ref10] Denis D. J. Sibilkov V. (2010). Financial constraints, investment, and the value of cash holdings. Rev. Financ. Stud. 23, 247–269. doi: 10.1093/rfs/hhp031

[ref11] Diebold F. X. Yilmaz K. (2009). Measuring financial asset return and volatility spillovers, with application to global equity markets. Econ. J. 119, 158–171. doi: 10.1111/j.1468-0297.2008.02208.x

[ref12] Du Y. Xie J. Chen J. (2019). CEO’s financial background and the Financialization of entity enterprises. China Ind. Econ. 5, 136–154. doi: 10.19581/j.cnki.ciejournal.2019.05.008

[ref13] Du Y. Zhang H. Chen J. (2017). The impact of Financialization on future development of real enterprises’ Core business: promotion or inhibition. China Ind. Econ. 12, 113–131.

[ref14] Earle J. S. Gehlbach S. (2015). The productivity consequences of political turnover: firm-level evidence from Ukraine's Orange revolution. Am. J. Polit. Sci. 59, 708–723. doi: 10.1111/ajps.12170

[ref15] Feng L. Fu T. Kutan A. M. (2019). Can government intervention be both a curse and a blessing? Evidence from China's finance sector. Int. Rev. Financ. Anal. 61, 71–81. doi: 10.1016/j.irfa.2018.10.010

[ref16] Hill M. D. Fuller K. P. Kelly G. W. Washam J. O. (2014). Corporate cash holdings and political connections. Rev. Quant. Finan. Acc. 42, 123–142. doi: 10.1007/s11156-012-0336-6

[ref17] Ho S. S. M. Wong K. S. (2001). A study of the relationship between corporate governance structures and the extent of voluntary disclosure. J. Int. Account. Audit. Tax. 10, 139–156. doi: 10.1016/S1061-9518(01)00041-6

[ref18] Jones B. F. Olken B. A. (2005). Do leaders matter? National leadership and growth since world war II. Q. J. Econ. 120, 835–864. doi: 10.1093/qje/120.3.835

[ref19] Kim C. Zhang L. (2016). Corporate political connections and tax aggressiveness. Contemp. Account. Res. 33, 78–114. doi: 10.1111/1911-3846.12150

[ref20] Krippner G. R. (2005). The financialization of the American economy. Soc. Econ. Rev. 3, 173–208. doi: 10.1093/SER/mwi008

[ref21] Krueger S. Walker R. W. (2008). Divided government, political turnover, and state bond ratings. Public Finance Rev. 36, 259–286. doi: 10.1177/1091142107309229

[ref22] Li H. Zhou L.-A. (2005). Political turnover and economic performance: the incentive role of personnel control in China. J. Public Econ. 89, 1743–1762. doi: 10.1016/j.jpubeco.2004.06.009

[ref23] Liu W. De Sisto M. Li W. H. (2021). How does the turnover of local officials make firms more charitable? A comprehensive analysis of corporate philanthropy in China. Emerg. Mark. Rev. 46:100748. doi: 10.1016/j.ememar.2020.100748

[ref24] Luo D. Chen K. C. Wu L. (2017). Political uncertainty and firm risk in China. Rev. Dev. Finance 7, 85–94. doi: 10.1016/j.rdf.2017.06.001

[ref25] Luo D. Liao J. Wang J. (2016). Local officials, turnover and corporate risk —— evidence from Chinese listed firms. Econ. Res. J. 51, 130–142.

[ref26] Opler T. Pinkowitz L. Stulz R. Williamson R. (1999). The determinants and implications of corporate cash holdings. J. Financ. Econ. 52, 3–46. doi: 10.1016/S0304-405X(99)00003-3

[ref27] Orhangazi Ö. (2008). Financialisation and capital accumulation in the non-financial corporate sector: a theoretical and empirical investigation on the US economy: 1973–2003. Camb. J. Econ. 32, 863–886. doi: 10.1093/cje/ben009

[ref28] Palley T. I. (2013). “Financialization: what it is and why it matters” in Financialization (Berlin: Springer), 17–40.

[ref29] Peng Y. Han X. Li J. (2018). Economic policy uncertainty and corporate Financialization. China Ind. Econ. 1, 137–155. doi: 10.19581/j.cnki.ciejournal.20180115.010

[ref30] Persson P. Zhuravskaya E. (2016). The limits of career concerns in federalism: evidence from China. J. Eur. Econ. Assoc. 14, 338–374. doi: 10.1111/jeea.12142

[ref31] Qian X. Xu Y. (2014). Official turnover, political identity and the risk taking of private listed firms. China Econ. Quart. 13, 1437–1460. doi: 10.13821/j.cnki.ceq.2014.04.009

[ref32] Shleifer A. Summers L. H. (2013). “2. Breach of Trust in Hostile Takeovers” in Corporate Takeovers: Causes and Consequences. ed. Auerbach A. J. (Chicago, IL: University of Chicago Press), 33–68.

[ref33] Sun Q. Tong W. H. S. (2003). China share issue privatization: the extent of its success. J. Financ. Econ. 70, 183–222. doi: 10.1016/S0304-405X(03)00145-4

[ref003] Stamboulis Y. Barlas A. (2014). Entrepreneurship education impact on student attitudes. Int. J. Manag. Educ. 12, 365–373. doi: 10.1016/j.ijme.2014.07.001

[ref34] Tori D. Onaran Ö. (2018). The effects of Financialization on investment: evidence from firm-level data for the UK. Camb. J. Econ. 42, 1393–1416. doi: 10.1093/cje/bex085

[ref36] Wang C. R. (2019). A literature review on corporate Financialization. Am. J. Ind. Bus. Manag. 9, 647–657. doi: 10.4236/ajibm.2019.93044

[ref37] Wang Q. Wen J. (2019). Local Officials' turnover and corporate innovation: the path of financing constraints and innovation contribution. Naikai Econ. Stud. 3, 198–225. doi: 10.14116/j.nkes.2019.03.011

[ref38] Wang X. Xu X. (2008). The source, whereabouts, tenure and economic growth of local officials—evidence from the secretary of the provincial party committee and the governor of China. Manag. World 3, 16–26. doi: 10.19744/j.cnki.11-1235/f.2008.03.003

[ref39] Xu N. Chen Q. Xu Y. Chan K. C. (2016). Political uncertainty and cash holdings: evidence from China. J. Corp. Finan. 40, 276–295. doi: 10.1016/j.jcorpfin.2016.08.007

[ref40] Xu X. Wang X. (2010). Promotion incentive and economic growth: evidence from Chinese provincial officials. J. World Econ. 33, 15–36.

[ref41] Zhang H. Li L. Zhou D. Zhou P. (2014). Political connections, government subsidies and firm financial performance: evidence from renewable energy manufacturing in China. Renew. Energy 63, 330–336. doi: 10.1016/j.renene.2013.09.029

[ref004] Zheng S. Kahn M. E. Sun W. Luo D. (2014). Incentives for China’s urban mayors to mitigate pollution externalities: the role of the central government and public environmentalism. Reg. Sci. Urban Econ. 47, 61–71. doi: 10.1016/j.regsciurbeco.2013.09.003

[ref43] Zhou L.-A. (2004). The incentive and cooperation of government officials in the political tournaments: An interpretation of the prolonged local protectionism and duplicative Investments in China. Econ. Res. J. 6, 33–40.

[ref44] Zhou L.-A. Liu C. Li X. Weng X. (2015). "layer upon layer" and official incentive. World Econ. Papers 1, 1–15.

[ref45] Zhu J. Zhang D. (2017). Does corruption hinder private businesses? Leadership stability and predictable corruption in China. Governance 30, 343–363. doi: 10.1111/gove.12220

